# Ligand-Assisted Purification of Mixed-Halide Perovskite Nanocrystals with Near-Unity PLQY for High-Color-Purity Display Applications

**DOI:** 10.3390/ma18214975

**Published:** 2025-10-31

**Authors:** Stephy Jose, Joo Yeon Kim, Hyunsu Cho, Chan-Mo Kang, Sukyung Choi

**Affiliations:** 1 Regional ICT Convergence Research Section, Greater Seoul Research Division, Electronics and Telecommunications Research Institute (ETRI), Seongnam-si 13488, Republic of Korea; stephymjo@etri.re.kr; 2 Advanced Materials and Devices Engineering, University of Science & Technology (UST), Daejeon 34113, Republic of Korea; 3 Reality Display Device Research Section, Electronics and Telecommunications Research Institute (ETRI), 218 Gajeong-ro, Yuseong-gu, Daejeon 34129, Republic of Korea; hyunsucho@etri.re.kr (H.C.); nkcm@etri.re.kr (C.-M.K.); skchoi915@etri.re.kr (S.C.)

**Keywords:** mixed-halide perovskite, near-unity PLQY, high-color-purity

## Abstract

Cesium halide perovskite nanocrystals (PNCs) have emerged as promising materials for application in high-color-purity displays due to their exceptional optoelectronic properties, which include narrow emission linewidths, tunable bandgaps, and high photoluminescence quantum yields (PLQYs). However, preserving these characteristics during purification remains a major challenge as surface ligand detachment during the washing process can lead to increased defect states, reduced quantum efficiency, and spectral broadening. The choice of anti-solvent plays a crucial role in maintaining the structural and optical integrity of PNCs, as it directly influences ligand retention and material stability. In this study, we propose an optimized purification strategy for mixed-halide perovskite nanocrystals that incorporates post-synthetic ligand supplementation, in which controlled amounts of oleic acid (OA) and oleylamine (OAm) are sequentially introduced into the crude solution prior to anti-solvent treatment. This approach reinforces surface passivation, suppresses trap state formation, and minimizes halide loss. Consequently, a near-unity PLQY with narrow full-width-at-half-maximum emissions is achieved for both green- and red-emissive nanocrystals, markedly enhancing color purity and providing a promising route toward next-generation wide-color-gamut display technologies.

## 1. Introduction

Cesium halide perovskite nanocrystals (PNCs) have emerged as a highly promising class of colloidal materials due to their remarkable optoelectronic properties including narrow emission spectra, high absorption coefficients, cost-effective solution processability, and tunable bandgaps across the visible spectrum [1,2,3,4,5]. The intrinsic versatility of the perovskite structure enables the incorporation of mixed halides, allowing for the precise modulation of optical and electronic properties such as bandgap energy, emission wavelength, and absorption characteristics. This tunability is particularly critical for high-color-purity display applications, including light-emitting diodes (LEDs) and next-generation display technologies, where spectral precision directly impacts visual performance.

For high-efficiency device fabrication, it is essential to isolate and purify as-synthesized PNCs without compromising their intrinsic properties. Due to their highly ionic nature, perovskite surfaces are typically passivated with organic ligands such as OA and OAm, which play crucial roles in maintaining structural integrity and colloidal stability [6,7,8]. However, the dynamic and unstable nature of these ligands often leads to their partial or complete detachment during purification, creating defect states and lowering the PLQY [9,10,11,12,13]. In severe cases, ligand loss triggers colloidal instability and irreversible aggregation. Such purification-induced ligand detachment and surface instability are not only detrimental to photoluminescence but also critically limit the operational lifetime and long-term stability of perovskite LEDs and color conversion layers [14,15]. These effects are further complicated by halide composition, as Br^−^ and I^−^ impart distinct surface chemistries and phase behaviors. Therefore, optimizing the washing protocol is essential to obtaining high-purity perovskite nanocrystals while preserving their desired properties.

Recognizing the pivotal role of purification in maintaining the optoelectronic integrity of PNCs, numerous studies have explored various washing methodologies to achieve an optimal balance between impurity removal and ligand retention. For instance, Li et al. [14] developed a purification strategy using a hexane–ethyl acetate solvent mixture to regulate the ligand density of CsPbBr_3_ quantum dots (QDs), demonstrating a significant enhancement in PLQY by carefully tuning the solvent polarity and perovskite ionicity. Similarly, Chiba et al. [12] refined ligand management techniques by employing butyl acetate (AcOBu) to selectively remove excess ligands from CsPbBr_3_ QDs, thereby improving both PLQY and charge injection properties in light-emitting diodes. Their subsequent work with low-dielectric solvents further highlighted the influence of the solvent dielectric constant on the precipitation and purification process of perovskites [11]. Meanwhile, Swarnkar et al. [16] proposed the use of methyl acetate (MeOAc) to stabilize the cubic phase of CsPbI_3_, effectively removing unwanted by-products while maintaining a high PLQY. Ligand-assisted purification strategies using a controlled combination of amines (oleylamine or dodecylamine) have also been applied to CsPbBr_3_, improving the photoluminescence quantum yield from 40% in conventionally purified nanocrystals to 83% with 5 vol% added oleylamine [17]. Although several studies have investigated purification strategies for preserving the optical properties of CsPbBr_3_ and CsPbI_3_ [10], a unified approach capable of enhancing the quantum yield of both green- and red-emissive perovskite materials remains to be overcome While previous studies have focused on post-synthetic ligand exchange to improve PLQY, our strategy stabilizes ligands in situ during anti-solvent washing, achieving near-unity PLQY for both green- and red-emissive perovskites [18,19]. Developing such a unified strategy is essential for the scalable manufacture of full-color displays where matched color purity, a high PLQY, and operational stability across all primary colors are required. In parallel, research on semi-transparent perovskite systems has underscored their importance for advanced displays and tandem architectures, where material purity and optical stability play pivotal roles [20].

To address this, we propose a novel washing process designed to decrease ligand detachment while simultaneously enhancing PLQY. Our approach involves the introduction of a small quantity of ligands before the addition of an anti-solvent during washing to improve ligand binding. By integrating ligand stabilization with anti-solvent purification, we successfully achieved near-unity PLQY for both green- and red-emissive mixed-halide perovskite. This method represents a significant advancement in the purification of perovskite nanocrystals, providing a scalable and effective approach for high-performance optoelectronic applications.

## 2. Experimental

### 2.1. Chemicals

Cesium carbonate (Cs_2_CO_3_, Aldrich, St. Louis, MO, USA, 99.9%), lead(II) bromide (PbBr_2_, TCI, Tokyo, Japan, 98%), lead(II) iodide (PbI_2_, Alfa Aesar, Heysham, UK, 99.9985%), 1-octadecene (ODE, Sigma-Aldrich, St. Louis, MO, USA, 90%), oleic acid (OA, Sigma-Aldrich, 90%), oleylamine (OAm, Sigma-Aldrich, 70%), tert-butanol (t-BuOH, St. Louis, MO, USA, ≥99.0%), hexane (anhydrous, St. Louis, MO, USA, 95%), and toluene (anhydrous, St. Louis, MO, USA, 99.8%).

### 2.2. Synthesis and Isolation of Mixed-Halide Perovskites

*Synthesis of Cs-oleate*: 2.5 mmol of Cs_2_CO_3_ (0.814 g) was weighed into a 100 mL three-neck flask. To this ODE (40 mL) and OA (2.5 mL) was added and vacuum-dried for 1 h at 110 °C with constant stirring, and then heated under a nitrogen environment until all of the Cs_2_CO_3_ reacted with the oleic acid. The clear cesium oleate solution was kept at 110 °C during injecting into the lead halide precursor.

*Synthesis of CsPbBr_3−x_I_x_ Perovskite NCs*: The main reaction flask was loaded with ODE (5 mL), OAm (0.5 mL), OA (0.5 mL), and a 0.188 mmol mixture of PbBr_2_ and PbI_2_; degassed for an hour at 110 °C; and then heated under a N_2_ environment to 165 °C. At this temperature, 0.4 mL of freshly prepared Cs-oleate solution (0.125 M in ODE) was rapidly injected over ~2–3 s. The reaction was arrested 30 s later by placing the mixture in an ice-water bath. To obtain red and green emissions, the amounts of PbBr_2_ and PbI_2_ were varied.

*Isolation and purification of CsPbBr_3−x_I_x_ Perovskite NCs*: In the standard purification protocol, a 1:1 volume ratio of ODE:tert-BuOH was employed [5]. The mixture was centrifuged at 15,000 rpm, the supernatant was discarded, and the precipitate was re-dispersed in hexane. In the modified purification process, equimolar OA and OAm (0.1 mL) were first introduced into the crude solution prior to the addition of tert-butanol. Under these conditions a reduced amount of *tert*-BuOH (3 mL) was sufficient to induce effective precipitation. Large amounts of butanol were found to excessively strip surface ligands, lowering the PLQY.

### 2.3. Fabrication of Perovskite Color Conversion Layer (CCL)

*Formulation of perovskite ink*: Green- and red-emissive mixed-halide perovskite nanocrystals (NCs) were individually dispersed in hexane to formulate perovskite resin inks. After solvent evaporation, the NCs were re-dispersed in toluene and mixed with a photo-curable resin (AD 1700, Solvay Solexix S.p.A, Bollate, MI, Italy) in a 1:1 weight ratio to obtain a homogeneous perovskite ink. The prepared ink was drop-cast onto blue OLED devices to fabricate CCLs, and the film thickness was controlled by adjusting the spin-coating speed. The resulting CCL thickness was measured using a surface profiler (α-step IQ, KLA-Tencor, Milpitas, CA, USA).

## 3. Characterization

TEM images were recorded using a JEOL JEM-2100FHR microscope (JEOL Ltd., Akishima, Japan) operated at 200 kV. Absorbance spectra were recorded using a Shimadzu UV3600 UV–Vis–NIR spectrometer (Shimadzu Corp., Kyoto, Japan) over the 300–800 nm range. Photoluminescence (PL) spectra were measured using an excitation wavelength of 375 nm. Photoluminescence quantum yield (PLQY) measurements were performed using an integrating sphere fluorescence spectrometer (Fluoro MateFS-2, Sinco, Daejeon, Republic of Korea) with an excitation wavelength of 450 nm. To minimize reabsorption and re-emission effects during PLQY measurements, the optical density of the samples was maintained at 0.05. Nuclear magnetic resonance (NMR) measurements were recorded on a Bruker Biospin (Billerica, MA, USA), Avance III HD Spectrometer operating at 400 MHz. The IV and luminance spectra of PNCs OLED devices were investigated using a source-measurement unit (Keithley-238, Keithley, Solon, OH, USA) and a spectroradiometer (CS-2000, Konica Minolta, Tokyo, Japan).

## 4. Results and Discussion

**Figure 1:**
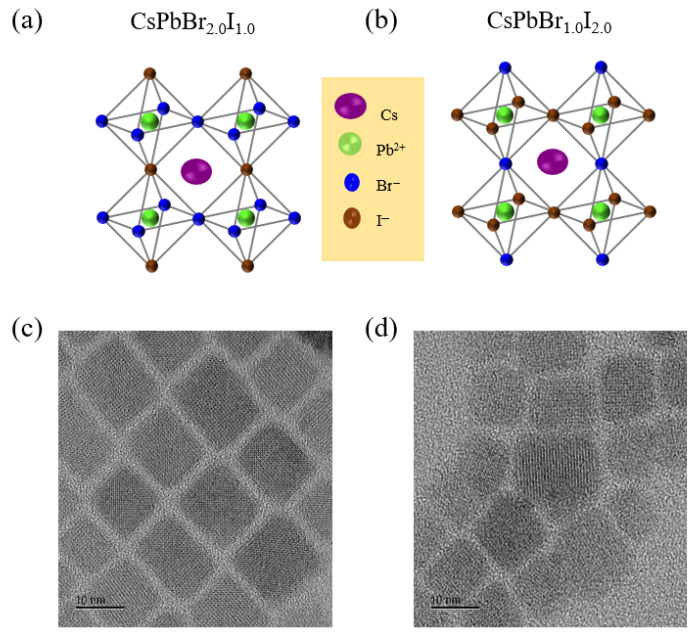
Schematics of the crystal structures of mixed-halide perovskite nanocrystals. (**a**) Green-emissive CsPbBr_2.0_I_1.0_, (**b**) red-emissive CsPbBr_1.0_I_2.0_, along with the corresponding TEM images of the synthesized nanocrystals: (**c**) CsPbBr_2.0_I_1.0_ and (**d**) CsPbBr_1.0_I_2.0_.

Green- and red-emissive mixed-halide PNCs with the general formula CsPbBr_3−x_I_x_ were synthesized via a hot-injection method by varying the molar ratio of halide ions (Br^−^: I^−^). For green-emissive perovskites, the Br^−^: I^−^ ratio was set at 2:1, corresponding to CsPbBr_2.0_I_1.0_, whereas red-emissive perovskites were obtained using a 1:2 ratio, resulting in CsPbBr_1.0_I_2.0_. The corresponding structures of these compositions are presented in Figure 1. Following synthesis and modified purification, the resulting PNCs were dispersed in hexane for subsequent characterization. TEM analysis confirmed that both green- and red-emissive mixed-halide PNCs exhibit a uniform cubic morphology. These nanocrystals also demonstrated high crystallinity and excellent dispersibility in nonpolar solvents such as hexane and toluene. The average particle size ranged from 10 to 12 nm, with CsPbBr_2.0_I_1.0_ displaying an average diameter of 10.2 nm, and CsPbBr_1.0_I_2.0_ measuring approximately 8.9 nm.

**Figure 2:**
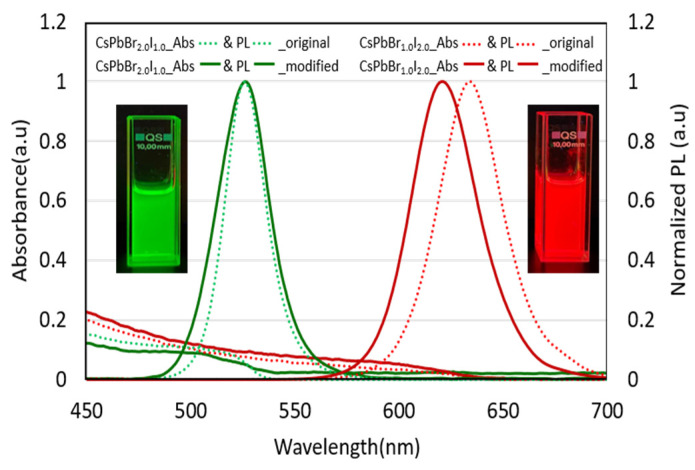
Absorption spectra and normalized PL spectra of green- and red-emissive PNCs purified using the original and modified washing processes. (Insets: Photographs of their emissions via the modified washing method under UV illumination).

To evaluate the optical properties, both green- and red-emissive mixed-halide PNCs were dispersed in hexane to form optically clear solutions with an optical density (OD) of approximately 0.05. As the molar fraction of iodide increased, both the absorption and photoluminescence (PL) spectra exhibited a noticeable redshift (Figure 2). This bathochromic shift is attributed to the incorporation of iodide ions, which are larger and less electronegative than bromide. The substitution of bromide with iodide in the perovskite lattice reduces the bandgap energy, thereby enabling emission tunability across the visible spectrum through halide composition engineering. While our results demonstrate effective emission tuning through halide composition, it is important to note that mixed-halide PNCs can undergo photo-induced phase segregation under continuous illumination [21]. This process produces iodine- and bromine-rich domains, which may further shift the emission and potentially compromise the long-term optoelectronic stability of the nanocrystals [22,23].

Table 1 summarizes the optical properties of mixed-halide PNCs purified using the original washing protocol versus the modified method. Green-emissive perovskites washed with tert-butanol (original method) exhibited a PL peak at 524.9 nm with a narrow emissive FWHM of 21.8 nm. However, the PLQY was limited to 26.91%, primarily due to the partial removal of surface ligands during washing with the polar anti-solvent tert-butanol.

These results are consistent with the findings reported by Hoshi et al. [9,11], who observed a significant decrease in the PLQY of CsPbBr_3_ when purified with polar solvents such as acetonitrile, acetone, and butanol (BuOH). Their study highlighted that BuOH, due to its high dielectric constant, is unsuitable for repeated washing cycles, as it promotes ligand desorption and induces halide vacancies, both of which contribute to the formation of non-radiative recombination centers and thus lower the PLQY. Interestingly, the red-emissive PNCs, which contained a higher molar ratio of iodide and were washed with BuOH, exhibited an improved PLQY of 61.52%, with a photoluminescence peak centered at 633 nm and a relatively narrow emission bandwidth of 36 nm. Despite the moderate quantum yield, the narrow FWHM of both green- and red-emissive PNCs purified using t-BuOH indicates their strong potential for high color purity in display applications.

However, for practical use in color conversion layers, further enhancement in terms of PLQY is essential. To this end, we introduced a modified washing strategy wherein small amounts of OA and OAm were added prior to the anti-solvent (t-BuOH) treatment, reporting near-unity PLQY values such as 99.68% only for green PNCs [17]. Therefore, here the modified washing strategies were introduced not only for green PNCs, but also for red PNCs and this approach has confirmed significantly improved the PLQY of both green- and red-emissive PNCs to near-unity levels, while preserving their narrow emission linewidths Notably, the green-emissive PNCs obtained via the modified washing protocol maintained an emission wavelength similar to their conventionally washed counterparts. In contrast, the red-emissive PNCs displayed a slight blueshift in their PL peak after the modified washing protocol. This shift is attributed to a reduction in particle size, which induces quantum confinement effects in a manner that is consistent with previous reports [5]. In addition, variations in halide content (e.g., Br^−^ vs. I^−^) can lead to significant differences in chemical stability, ligand–ion interactions, and phase behavior. Importantly, as shown by De Roo et al. [17], OA and OAm bind dynamically to CsPbX_3_ nanocrystal surfaces, and their continuous exchange modulates surface passivation and lattice structure. Given the different binding affinities of Br^−^ and I^−^, this dynamic environment can further induce composition-dependent lattice relaxation and surface dipole variations, thereby explaining the distinct emission peak shifts observed for CsPbBr_2_I and CsPbBrI_2_ after OA/OAm treatment. These differences necessitate a more tailored purification strategy that considers the halide-dependent surface chemistry of PNCs to minimize degradation and maximize optical performance.

In this study, we retained tert-butanol as the anti-solvent in the modified washing process to leverage its effectiveness at reprecipitating PNCs from the crude reaction mixture and efficiently removing unreacted precursors and soluble impurities. However, it is well established that ionic PNCs are highly sensitive to polar solvents, including alcohols such as tert-butanol. Prior studies have reported that washing with polar anti-solvents can lead to a reduction in the I/Br ratio, primarily due to the preferential removal of surface-bound iodide ions [9,23,24,25]. This phenomenon may arise from differences in the solubility of halides in polar solvents or the relatively weaker binding affinity between iodide and Pb^2+^ compared to bromide.

Despite these limitations, the inclusion of an anti-solvent step remains essential to achieving effective purification. To overcome the detrimental effects of ligand displacement and halide loss during anti-solvent treatment, developing a modified purification strategy that enhances PLQY while maintaining structural integrity is critical. Anti-solvents with high dielectric constants readily disrupt ligand–surface interactions as the binding of organic ligands to the perovskite surface is highly dynamic and reversible [24,26]. This displacement can lead to the removal of halide anions from the [PbX_6_]^4−^ octahedral framework, resulting in the generation of uncoordinated Pb^2+^ ions. These undercoordinated lead sites act as trap states that facilitate non-radiative recombination, significantly reducing PLQY.

Therefore, to suppress trap formation and enhance optical performance, it is necessary to increase the surface ligand density, thereby reinforcing halide binding and passivating undercoordinated Pb^2+^ ions. The modified washing approach presented in this work addresses these challenges by introducing a small amount of ligands prior to anti-solvent addition, resulting in improved surface passivation and a higher PLQY.

CsPbBr_3_ is known to maintain a stable cubic crystal phase across a wide range of solvents, making it compatible with various anti-solvents such as methanol, acetonitrile, and 1-butanol [9,10]. This bromide-based, green-emissive material exhibits relatively strong ligand binding, which allows anti-solvent washing to effectively remove excess ligands without significantly compromising structural integrity or optical performance.

In contrast, CsPbI_3_ demonstrates a weaker interaction between its surface and organic ligands due to the comparatively softer basic nature of iodide (I^−^) relative to bromide (Br^−^). This leads to a reduced acid–base interaction between I^−^ and the oleylammonium (~NH_3_^+^) group of OAm ligands. As a result, CsPbI_3_ tends to undergo rapid ligand desorption, agglomeration, and an unfavorable phase transition from the optically active cubic phase to the non-emissive orthorhombic phase [6,10,17,27,28].

This disparity in ligand binding strength between CsPbBr_3_ and CsPbI_3_ not only affects the structural stability of the nanocrystals but also significantly influences their PLQY. Since facile ligand detachment is a primary cause of increased surface trap density and reduced PLQY, introducing an excess amount of oleylamine (OAm) during synthesis or purification serves as an effective strategy to ensure sufficient oleylammonium (OAM) coordination for surface defect passivation. This additional ligand stabilization improves both the optical stability and emission efficiency of iodide-rich perovskite nanocrystals [10,17]. De Roo et al. demonstrated that the addition of excess surface ligands during the purification process can effectively preserve the optical, colloidal, and structural integrity of CsPbBr_3_ PNCs. Furthermore, their study showed that increasing the concentration of amine-containing ligands led to an enhancement in PLQY [17].

**Figure 3:**
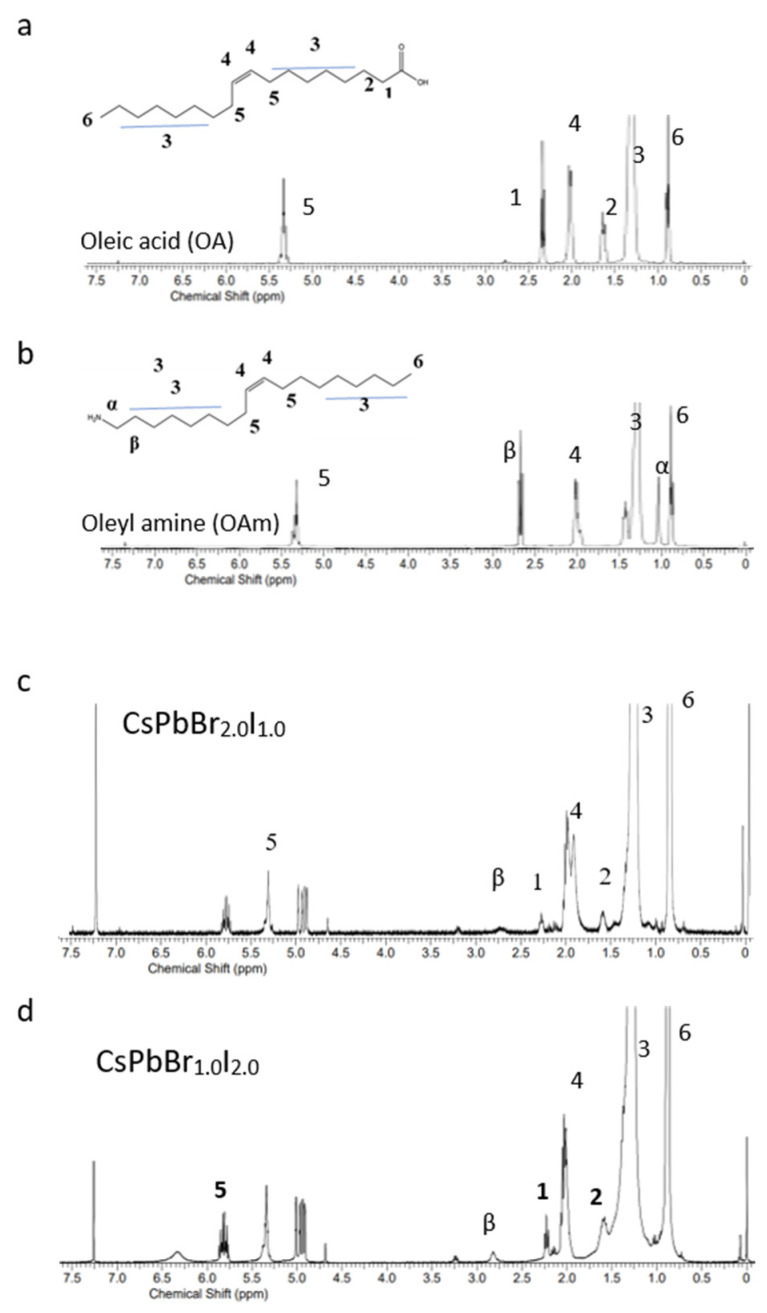
^1^H-NMR spectra of (**a**) oleic acid (OA)and (**b**) oleylamine (OAm) ligands for comparison as a reference, (**c**) green-emissive CsPbBr_2.0_I_1.0_ and (**d**) red-emissive CsPbBr_1.0_I_2.0_ PNCs obtained using the ligand-assisted modified washing method. Resonances α and β correspond to the NH^3+^ and α-CH_2_ groups of bound ligands, respectively.

Inspired by these findings, we introduced a small amount of OA and OAm into the crude perovskite solution prior to the addition of tert-butanol during the washing step. This approach was designed to improve ligand binding on the PNCs’ surface, thereby enhancing stability and emission efficiency. The effectiveness of this strategy was confirmed by proton nuclear magnetic resonance (^1^H-NMR) spectroscopy. Specifically, the broad resonances corresponding to the α-CH_2_ group of oleylammonium (Resonance β in Figure 3) and the NH_3_^+^ group (Resonance α in Figure 3) indicate successful ligand binding. The broad NH^3+^ resonance suggests the efficient protonation of OAm during surface coordination, which is consistent with previous reports [6].

In the case of green-emissive CsPbBr_2.0_I_1.0_ PNCs, these features confirm strong ligand binding, correlating with the observed near-unity PLQY of 99.68%. In contrast, red-emissive CsPbBr_1.0_I_2.0_ PNCs exhibited notably broader and weaker resonance signals, indicating less effective ligand coordination. This difference is attributed to the weaker acid–base interaction between I^−^ and the oleylammonium (~NH_3_^+^) group, which leads to a reduced ligand binding strength. Consistent with reported computational studies, our experimental results indicate that the binding strength of oleylammonium (OAM) ligands decreases with increasing iodide content, leading to the weaker surface passivation and reduced stability and photoluminescent performance of I-rich nanocrystals.

**Figure 4:**
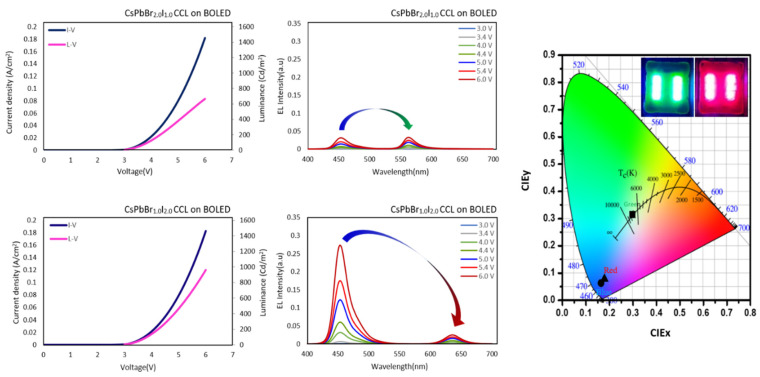
EL spectra illustrating the color conversion efficiency of green-emissive (CsPbBr_2.0_I_1.0_) and red-emissive (CsPbBr_1.0_I_2.0_) PNCs-based color conversion layers (CCLs), fabricated via ligand-assisted modified washing, measured at an operating voltage of 6.0 V. The spectra demonstrate the relative intensities of converted green and red emissions, along with residual blue emission leakage from the underlying blue OLED device. (Inset photo: green- and red-converted emissions on a BOLED operated at 6 V).

To demonstrate the practical utility of these nanocrystals, we fabricated green- and red-emissive perovskite color conversion layers (CCLs) on the BOLED, as shown in Figure 4. The synthesized green- and red-emissive PNCs were first dispersed in anhydrous hexane, then re-dispersed in toluene to ensure compatibility with the UV-curable resin used for ink formulation. A commercial resin, Fluoro Ink AD 1700, was used to prepare a homogeneous perovskite ink. This ink was subsequently spin-coated onto the BOLED substrate to form CCLs with thicknesses of approximately 2–2.5 μm, as measured by alpha step, thereby demonstrating the feasibility of these materials in high-color-purity display applications. The electroluminescence spectra of the CsPbBr_2.0_I_1.0_ CCL showed narrow green emissions with the highest intensity at 6.0 volts, while the CsPbBr_1.0_I_2.0_ CCL also exhibited narrow red emissions at the same voltage. Although both layers exhibited some blue leakage, the red CCL showed higher blue leakage, which can be attributed to its lower PLQY compared to the green-emissive PNCs. Figure 4 presents the CIE 1931 chromaticity diagram of the perovskite-based CCLs. Although the observed emissions appear visibly green and red, the measured chromaticity coordinates deviate slightly from the ideal regions, indicating the need for further optimization to improve the color conversion characteristics. These findings serve as a proof-of-concept demonstration, underscoring the potential applicability of the synthesized nanocrystals in display technologies and establishing a foundation for future research aimed at enhancing color purity and overall device performance.

## 5. Conclusions

In summary, this study presents a modified washing protocol for the purification of colloidal CsPbBr_3−x_I_x_ PNCs with green and red emission. The protocol involves the strategic addition of a small amount of oleic acid and oleylamine prior to anti-solvent treatment with tert-butanol. This approach effectively mitigates ligand detachment during purification, resulting in nanocrystals with near-unity PLQYs while maintaining spectrally narrow emission profiles.

Compared to conventional washing methods using polar solvents alone, which often result in significant ligand loss and reduced emission efficiency, our results indicate that combining polar anti-solvents with pre-added OA/OAm ligands preserves the structural and optical integrity of the perovskite NCs. Moreover, the practical applicability of these materials was demonstrated through color conversion layers fabricated on blue OLED substrates, underscoring their potential for use in high-color-purity display technologies. Importantly, the proposed protocol relies on straightforward, solution-based steps employing common ligands and solvents, suggesting that it can be readily scaled for industrial production while maintaining nanocrystal stability and optical performance. These findings emphasize the crucial role of surface ligand engineering in perovskite nanocrystal purification and offer a robust, scalable route toward the production of high-performance, compositionally tunable emissive materials for next-generation optoelectronic devices.

## Figures and Tables

**Table 1:** Comparison of optical properties (emission wavelength, FWHM, and PLQY) of green- and red-emissive CsPbB_3−x_I_x_ perovskite nanocrystals purified by the original and ligand-assisted modified washing methods.

PNCs		PL Max (nm)	FWHM (nm)	PLQY (%)
CsPbBr_2.0_I_1.0_	Original	524.9	21.8	26.91
	Modified	525.9	23.1	**99.68**
CsPbBr_1.0_I_2.0_	Original	633.4	36.0	61.52
	Modified	620.3	37.7	**93.12**

## Data Availability

The original contributions presented in this study are included in the article. Further inquiries can be directed to the corresponding author.
